# Cryoneurolysis Is a Safe, Effective Modality to Improve Rehabilitation after Total Knee Arthroplasty

**DOI:** 10.3390/life12091344

**Published:** 2022-08-29

**Authors:** Brandon E. Lung, Theofilos Karasavvidis, Abhinav K. Sharma, Arya Amirhekmat, Hayk Stepanyan, William McMaster, Steven Yang, David H. So

**Affiliations:** Department of Orthopaedic Surgery, School of Medicine, University of California, Irvine, CA 95616, USA

**Keywords:** total knee arthroplasty, cryoneurolysis, postoperative pain control

## Abstract

Although long term pain and mobility outcomes in total knee arthroplasties (TKA) are successful, many patients experience significant amount of debilitating pain during the immediate post-operative period that necessitates narcotic use. Percutaneous cryoneurolysis to the infrapatellar saphenous and anterior femoral cutaneous nerves may help to better restore function and rehabilitation after surgery while limiting narcotic consumption. A retrospective chart review of primary TKA patients receiving pre-operative cryoneurolysis from 2019 to 2020 was performed to assess total opioid morphine milligram equivalents (MME) consumed inpatient and at interval follow-up. Demographics and medical comorbidities were compared between cryoneurolysis and age-matched control patients to assess baseline characteristics. Functional rehabilitation outcomes, including knee range of motion (ROM), ambulation distance, and Boston AM-PAC scores, as well as patient reported outcomes using the KOOS JR and SF-12 scores were analyzed using STATA 17 Software. The analysis included 29 cryoneurolysis and 28 age-matched control TKA patients. Baseline demographics and operative technique were not significant between groups. Although not statistically significant, cryoneurolysis patients had a shorter length of stay (2.5 vs. 3.5 days) and overall less inpatient and outpatient MME requirements. Cryoneurolysis patients had statistically significant improved 6-week ROM and 1-year follow-up KOOS JR and SF-12 mental scores compared to the control. There were no differences in complication rates. Cryoneurolysis is a safe, effective treatment modality to improve active functional recovery and patient satisfaction after TKA by reducing MME requirements. Patients who underwent cryoneurolysis had on average fewer MME prescribed during the perioperative period, improved active ROM, and improved patient-reported outcomes with no associated increased risk of infections, deep vein thrombosis, or neurologic complications.

## 1. Introduction

Total knee arthroplasty (TKA) is a highly successful and cost-effective procedure for chronic knee osteoarthritis, with a 10-year survival of 94% and 20-year survival of 71% according to recent literature [1]. Though TKA is a well-established option for improving patients’ quality of life through pain alleviation, restoration of function and range of motion, and correction of deformity, patient satisfaction has been less predictable with reports of up to a 20% dissatisfaction rate [2,3,4,5,6,7]. Furthermore, this rate of dissatisfaction has remained static despite significant improvements in surgical technique, biomaterials, implant designs, and technology [3,8]. With a projected exponential increase in the number of TKAs performed in the coming decades, it is important for arthroplasty surgeons to continue to understand drivers of patient dissatisfaction and attempt to address them before, during, and after surgery [9].

One of the most significant predictors of postoperative dissatisfaction following TKA is a painful total knee replacement [2]. Factors such as patients’ preoperative expectations, residual symptoms, tourniquet use, and mental health scores have all been shown to have considerable effects on patients’ perception of pain and satisfaction following surgery [2,5,10,11]. Opioids are a significant component of perioperative pain management, with studies demonstrating up to one-third of opioid naïve TKA patients using opioid medications at three months postoperatively for pain control, and even more in the case of those consuming opioid medications prior to surgery [12]. With an ongoing opioid consumption crisis in the United States, with reports of over 30,000 opioid-related U.S. deaths in 2015, it is critical for orthopaedic surgeons to consider a multimodal pain strategy for patients to minimize opioid use and decrease risk of opioid use dependence following surgery [13,14,15]. Not only are opioids not recommended for or against by the American Association of Orthopaedic Surgeons, chronic opioid use is correlated with worse postoperative functional outcomes and increased morbidity and mortality [13,16,17].

Cryoanalgesia (cryoneurolysis) has emerged as one such method to manage knee pain and reduce persistent postoperative pain in patients undergoing TKA. This procedure entails the induction of neuronal degeneration through exposure of nerve endings to temperatures below −20 °C and functions similar to a long-active nerve block [18,19,20]. In TKA, the superficial genicular nerves (infrapatellar branches of the saphenous nerve, ISN, and anterior femoral cutaneous nerve, AFCN) are targeted to reduce pain over the anterior aspect of the knee [18]. Recent literature have reported the promise of this modality for reducing postoperative pain, with randomized-controlled studies of 62, 100, and 121 patients demonstrating a significant correlation between preoperative cryoneurolysis and less pain, fewer opioid prescriptions, and improved functional outcomes [18,21,22].

In this study, we sought to validate these findings through assessing the efficacy and safety of cryoneurolysis treatment prior to TKA, with a specific focus on evaluation of postoperative knee range of motion and opioid consumption. Our hypothesis was that preoperative cryoneurolysis would result in increased postoperative knee range of motion and decreased stiffness as well as decreased postoperative opioid consumption.

## 2. Materials and Methods

### 2.1. Study Design

This study is a retrospective cohort analysis comparing patients receiving cryoneurolysis prior to TKA against controls undergoing TKA without cryoneurolysis. All information was abstracted from patients undergoing standard of care TKA. Data from patients’ care was obtained from the pre-operative course through completion of follow up. The primary outcomes of the study were range of motion improvement and opioid analgesics use reported in milligram morphine equivalents (MME). The secondary outcomes were any kind of complication after surgery. The study was performed according to the strengthening the reporting of observational studies in epidemiology (STROBE) guidelines [23].

### 2.2. Patient Population and Ethical Considerations

A retrospective chart review of 57 patients, with diagnosed knee osteoarthritis, who underwent TKA by a fellowship-trained orthopaedic surgeon (DS) between 2019 and 2020 was performed. Twenty-nine patients had undergone cryoneurolysis 1 week prior to TKA. Twenty-eight age-matched patients who were not treated with cryoneurolysis before TKA were used as a control group. Patients who presented any additional knee pathology, below the age of 18, and pregnant were excluded from our study. The study protocol was reviewed and approved by the University of California, Irvine Institutional Review Board, and patient informed consent was waived due to the retrospective design of the study.

### 2.3. Data Abstraction

Demographic variables included age, sex, body mass index [BMI], diabetes, smoking, ASA status, preoperative lab values, history of prior knee surgery or chronic pain, and opioid use before surgery. Perioperative variables obtained included tourniquet time, adductor canal block, use of a continuous nerve catheter, blood loss, inpatient MME intake, length of hospital stay, distance walked on discharge, and Boston AM-PAC scores. Inpatient MME was calculated using the total dose of opioid analgesics administered normalized to MME. Total distance walked was a summation of the reported patient distance traveled with physical therapy teams on postoperative day 1. The postoperative outcomes variables collected were 4–6-week MME prescribed, clinical complications, and range of motion as reported by the treating providers. Patient reported outcome variables (PROM) included subjective pain and functional outcome scores as outlined in Knee Injury and Osteoarthritis Scores (KOOS JR) and Short Form Survey (SF 12), which have been validated by previous studies [18,21,24]. Outpatient MME was calculated using total doses of opioid analgesics prescribed by the treating physicians normalized to MME. Patients whose pain regimen was managed by a third-party pain specialist or had missing data were excluded.

### 2.4. Cryoneurolysis Technique

Cryoneurolysis was administered using the Iovera device by a fellowship trained sports medicine physician 1 week before planned TKA. The AFCN and ISN were targeted using the technique as previously described by Dasa et al. Treatment lines for each corresponding nerve consisted of 6 treatment cycles, each lasting about 60 s, and a 90 mm SmartTip probe was used to administer the cryoneurolysis with direct patient feedback.

### 2.5. Statistical Analysis

Continuous variables were described with mean ± standard deviation or mean and range, whereas categorical variables were reported with absolute and relative frequency [25]. The Wilcoxon rank-sum test was conducted to compare continuous variables, while binary outcomes were compared using the Chi-square or Fisher exact test as appropriate. For all tests, *p* < 0.05 was considered statistically significant. Stata 17 (StataCorp LLC, College Station, TX, USA) was used as statistical software for all analyses.

## 3. Results

The study population had a mean age of 67–68 years, and there were no significant differences in ASA, medical comorbidities, gender, and prior history of chronic pain between the groups, which reflects the validity of the age and comorbidity matching (Table 1). Although perioperative data demonstrated a significantly higher blood loss in the cryoneurolysis group relative to the controls (100 ± 72 vs. 64 ± 44 *p* = 0.0463), there was no difference in infections, deep vein thrombosis, discharge destination, cardiac complications, or length of stay between groups (Table 2). All other perioperative metrics, including tourniquet time, showed no differences, suggesting that both groups underwent TKA in a similar fashion.

The primary outcome of MME consumed showed no statistically significant differences during the inpatient stay as well as clinic visits (Table 3). Despite not reaching significance, the averages of the cryoneurolysis group compared to the control appeared lower at the 4-week postoperative visit (313 ± 478 vs. 561 ± 619 *p* = 0.0534). 6-week ROM improvements relative to pre-operative measurements in the treatment group reached statistical significance (12 ± 9 vs. 3 ± 12 *p* = 0.042), although earlier interval measurements were not significant (Table 4). While not statistically significant, cryoneurolysis patients on average ambulated 100 feet farther than the treatment group on postoperative day one.

Patient reported outcomes demonstrated a statistically significant increase in the improvement in KOOS scores from pre-op to 12 months (38.8 (11.2) vs. 11.1 (9.6) *p* = 0.007) (Table 5). There was also a significantly greater reported SF 12 mental score at 12 months (60.4 (5.1) vs. 50.4 (6.7) *p* = 0.01). All other patient reported outcomes, including VAS inpatient pain scores and earlier interval KOOS JR and SF 12 mental and physical scores, failed to reach significance.

## 4. Discussion

Cryoneurolysis is a safe, effective preoperative modality that can be incorporated into an integrated multimodal pain management pathway to reduce opioid consumption and improve knee functional outcomes. Management of pain is an important part of patient care and satisfaction, and identifying techniques to reduce postoperative opioid dependence improves the physician-patient communication on expected outcomes and discharge planning. Previous studies have found prolonged postoperative use of opioids after TKA to be associated with increased rates of stiffness, poor functional outcomes, and limitations on quality of life [26]. Implementation of multimodal regimens, including nonsteroidal anti-inflammatory drugs, gabapentinoids, acetaminophen, periarticular injections, and neuroaxial analgesia have been previously explored to reduce opioid consumption without compromising rehabilitation strength [27]. However, oral medications and periarticular injection modalities allow for relatively short temporary relief, whereas cryoneurolysis therapy may provide sustained analgesia many weeks postoperatively [21]. this retrospective review, we assess for inpatient and outpatient opioid consumption, subjective functional outcome scores, and rehabilitation mobility as assessed by a physical therapist in order to understand the dynamics of cryoneurolysis on short and long-term recovery. Overall, patients who underwent cryoneurolysis had on average fewer opioid requirements during the perioperative period, improved active range of motion, and improved patient-reported outcomes with no associated increased risk of infections, deep vein thrombosis, or neurologic complications.

Despite not reaching statistical significance, cryoneurolysis patients required on average less MMEs compared to the control group both in the acute inpatient stay and postoperative discharge period. The decreased MME requirement is clinically significant since fewer opioids overall decreases the risk of respiratory complications, falls, nausea, vomiting, constipation, urinary retention, and cognitive impairment, which are side effects that can significantly increase the length of stay, prolong the rehabilitation process, and increase hospital costs [28]. In fact, cryoneurolysis patients on average were discharged 1 day sooner than the control group, which is clinically significant as the extended stay is important to consider from a billing, hospital bed space, and hospital quality metrics perspective [29]. With the emphasis on value-based bundled healthcare, it is important for surgeons to identify ways to shorten length of stay to reduce risks for nosocomial infections and hospital complications.

Prolonged opioid use after TKA has been associated with increased rates of stiffness, infection, and poor satisfaction scores, and our cryoneurolysis patients at the 4–6 weeks clinic visit had nearly half the prescribed amount of opioids compared to the control group [26]. While not statistically significant, the overall reduced opioids prescribed 1 month after surgery compared to the control group suggest clinically improved pain perception that reduces unnecessary health care utilization of emergency rooms and office phone visits for unrelieved pain [30]. The decrease in overall narcotics prescribed both inpatient and outpatient are reaffirmed through our anecdotal reports by hospital and physical therapy staff that report greater satisfaction and improved functional rehabilitation in our cryoneurolysis patients.

In terms of active recovery and return to independence, patients in the cryoneurolysis group ambulated on average 100 feet farther than the control group on postoperative day 1 despite not reaching statistical significance. The improvement in ambulation on postoperative day 1 may reflect the improved endurance and strength to participate in physical therapy, which oftentimes may be limited by confounding effects of nausea, vomiting, and sedation from high opioid intake. Overall reduced opioid intake combined with improved pain control may contribute to greater participation during therapy sessions that lead to earlier and safer discharge to home [30]. Although the Boston AM-PAC scores, which evaluate amount of dependence needed to complete activities of daily living, were not significantly improved in the treatment group, day 1 ambulatory improvements may better represent the effectiveness of cryoneurolysis on quadriceps coordination needed for rapid rehabilitation [31]. The Boston AM-PAC scores evaluate tasks such as bed mobility and upper body grooming, which may not necessarily reflect functional recovery after TKA.

In fact, regaining knee ROM is an important postoperative outcome that has been shown in the literature to affect ability to return to activities of daily living, such as going up and down stairs and getting up from a chair [32]. At the 6-week postoperative clinic visit, cryoneurolysis patients had statistically significant improved degrees of knee motion compared to preoperative measurements, which suggest better motion of the knee joint that is not as limited by postoperative pain and muscle spasms [33]. Recovery of knee ROM is non-linear, and cases of insufficient early recovery of motion have resulted in permanent dysfunction, revision surgery, and poor satisfaction [34]. In our study, by the 6 week visit, the cryoneurolysis patients had on average a 12 degree improvement of ROM compared to before surgery, which may reflect better short-term rehabilitation, less pain-limiting motion, and faster return to activity. Cryoneurolysis may be an effective modality to regain and improve knee ROM especially in cases where patients may be hesitant to move their knees due to pain and risk developing stiffness requiring return to OR [24].

With the emphasis on pay for performance measures to evaluate TKA outcomes, PROMs, such as the KOOS Jr. and SF-12, have become widely used in the literature to gauge patient satisfaction and knee function [35]. Similar to other studies, cryoneurolysis patients reported improved mean gain of function and quality of life especially at the 1-year follow-up compared to the control group [21]. At both the 3 month and 1-year visit, cryoneurolysis patients achieved KOOS Jr mean clinically important differences, which may suggest the effectiveness of cryoneurolysis on reducing long term knee pain and difficulty associated with daily living [36]. Not only was functional mobility reported to have improved, but also quality of life assessment as measured by the SF-12 mental had a greater impact in the cryoneurolysis patients compared to the control at 1 year. SF-12 mental has been used as a diagnostic tool for depression, and improved overall scores in the cryoneurolysis group may be a reflection of reduced opioid consumption, which has been previously shown to cause long-term catastrophic thinking and symptoms of depression and post-traumatic stress disorder [37].

However, there are limitations to consider in this study, including its retrospective design, nonrandomized nature, and lack of blinding of patients which may limit the generalizability of our results. Due to the relatively new implementation of the technique performed in a single surgeon cohort, there were limited number of patients in the analysis and many patient-reported outcome measures were not fully completed. It is possible that many outcomes deemed not statistically significant may not have been powered to elucidate associations due to the small sample size. As there was no phone call or recorded measurement of pain scores one day prior to surgery, we were unable to assess the interval change in patient’s symptoms from time between undergoing cryoneurolysis and surgery. In addition, although outpatient opioids prescribed may have been used for other sources of pain and may not truly reflect the actual use of the narcotic, prescribed amounts of MME at clinic visits were used in this study as reflective of opioid requirements.

Overall, cryoneurolysis is a safe treatment with no evidence of dyesthesias or numbness complications reported to interfere with patient rehabilitation in this study. While other studies report neuropathic pain, deep vein thrombosis, and wound infections associated with the surgical incision to be possible limitations to cryoneurolysis, this study found no increased risk compared to the control group [26]. Although cryoneurolysis patients had a statistically significant increased surgical EBL, this finding is likely an artifact due to the sample size and not attributable to cryoneurolysis, as previous studies have also found no increased risk of vascular damage or dermal bleeding from the treatment [22,38]. There were no instances of prolonged sensory or motor proprioception block that increased the risk of falling or interfered with therapy participation.

## 5. Conclusions

Cryoneurolysis is a safe, effective treatment modality for patients undergoing TKA to reduce opioid consumption, improve knee ROM, and improve patient satisfaction. Although there was no statistically significant reduction in MME consumed between groups, there was a trend towards reduced MME prescribed at follow-up visits. The improved knee ROM at 6-week follow up evaluations in the cryoneurolysis group may reflect a more active recovery process and faster return to activities of daily living.

## Figures and Tables

**Table 1:** Baseline demographic and clinical characteristics.

Variables	Cryoneurolysis (*n* = 29) (%)	Control (*n* = 28) (%)	*p* Value
Age	68 ± 7	67 ± 7	0.70
Left side	15 (51.7)	10 (35.7)	0.28
Female	19 (65.5)	18 (64.3)	0.92
BMI	33 ± 5	31 ± 6	0.10
Diabetes	8 (28.5)	8 (30.7)	0.86
Smoking	3 (10.7)	2 (7.7)	0.70
Steroids	1 (3.5)	0	1.00
ASA			0.66
II	13 (54.1)	12 (48)	
III	11 (45.8)	13 (52)	
Pre-op Hb	13.2 ± 1.4	14.1 ± 1.7	0.07
INR	1.01 ± 0.19	0.98 ± 0.08	0.85
Albumin	4.1 ± 0.5	4.3 ± 0.3	0.07
HbA1c	6.7 ± 1.3	6.4 ± 1.1	0.82
MI	1 (3.7)	2 (7.7)	0.61
CHF	1 (3.7)	0	1.00
PVD	2 (7.4)	2 (7.7)	0.96
CVA	0	2 (7.7)	0.23
Dementia	0	1 (3.8)	0.49
COPD	1 (3.7)	3 (11.5)	0.35
CKD	3 (11.1)	2 (7.7)	0.67
Ca any	4 (14.8)	2 (7.7)	0.66
Liver Disease	1 (3.7)	0	1.00
AIDS	0	1 (3.8)	0.49
Hx of any arthroplasty	5 (18.5)	4 (15.4)	0.76
Hx of prior knee surgery	1 (3.7)	4 (15.4)	0.19
Hx of chronic pain	11 (40.7)	10 (38.4)	0.86
Opioid use before sx	5 (18.5)	5 (19.2)	0.94
Coagulopathy	3 (11.1)	3 (11.5)	0.96

**Table 2:** Perioperative characteristics.

Variables	Cryoneurolysis (*n* = 29) (%)	Control (*n* = 28) (%)	*p* Value
Tourniquet time	75 ± 11	86 ± 24	0.24
Length of stay	2.5 ± 0.8	3.5 ± 2.9	0.12
Adductor canal block	25 (92.6)	25 (92.6)	1.00
Postop continuous nerve catheter	6 (22.2)	2 (7.4)	0.12
MME inpatient (median, range)	29.3 ± 23.8 (24.3, 0–86.5)	35 ± 24 (25, 1.2–83)	0.31
Acute Pain Service inpatient consult	4 (15.3)	3 (12)	0.72
Distance walked on discharge (median, range)	154 ± 119 (135, 50–600)	193 ± 121 (180, 45–500)	0.0640
Postop DVT ppx			0.49
ASA	25 (92.6)	24 (100)	
Enoxaparin	2 (7.4)	0	
DVT/PE	0	0	
Estimated blood loss (median, range)	100 ± 72 (75, 25–300)	64 ± 44 (50, 5–200)	**0.0463**

**Table 3:** 6-week outcomes.

Variables	Cryoneurolysis (*n* = 29) (%)	Control (*n* = 28) (%)	*p* Value
MME (median, range)			
4 weeks	313 ± 478 (88, 0–2025)	561 ± 619 (450, 0–2565)	0.0534
6 weeks	154 ± 427 (0, 0–2025)	289 ± 439 (0, 0–1350)	0.19
Surgical site infection	0	1 (3.7)	0.31
Pulmonary complication	0	1 (3.8)	1.00
Cardiac complication	1 (3.8)	1 (3.8)	1.00
Neuro complication	0	0	

**Table 4:** Range of motion.

Variables	Cryoneurolysis	Control	*p* Value
Preop	102 ± 14	109 ± 18	0.14
Postop			
2 weeks	88 ± 13	91 ± 14	0.71
4 weeks	106 ± 9	109 ± 13	0.31
6 weeks	112 ± 10	116 ± 11	0.40
6-weeks improvement (median, range)	12 ± 9 (10, −10 to 35)	3 ± 12 (5, −25 to 25)	**0.0420**

**Table 5:** Post Acute Care (PAC), Knee Injury and Osteoarthritis Outcome Score (KOOS), and Short Form Survey 12 (SF12) scores.

Score	Cryoneurolysis Mean (SD)	Control Mean (SD)	*p* Value
PAC	17.3 (3.2)	17.5 (2)	0.8
Preop KOOS	15.7 (4.8)	17.9 (6.4)	0.2
3mo KOOS	8.1 (5.9)	10 (4.8)	0.5
3mo KOOS difference	−0.8 (13.8)	−10 (8.3)	0.4
12mo KOOS	5.7 (7.3)	7.7 (2.5)	0.1
12mo KOOS difference	−11.2 (6.1)	−7 (6.5)	0.2
Preop KOOS interval	47.4 (13.7)	40.7 (18.6)	0.2
3mo KOOS interval	66.4 (14.8)	62 (11.9)	0.5
3mo KOOS interval difference	27.5 (10)	25.7 (22.1)	0.4
12mo KOOS interval	77 (21.7)	65.7 (5.5)	0.1
12mo KOOS interval difference	38.8 (11.2)	11.1 (9.6)	**0.007**
Preop SF12 physical	33.1 (9.3)	32.4 (9)	0.8
3mo SF12 physical	38.3 (8.7)	33.3 (12.1)	0.4
3mo SF12 physical difference	8.8 (4.3)	2.5 (18.2)	0.1
12mo SF12 physical	45 (11.5)	43.2 (6.6)	0.4
12mo SF12 physical difference	12.9 (11.4)	4 (7.8)	0.2
Preop SF12 mental	54.5 (7.9)	51.2 (10.7)	0.4
3mo SF12 mental	53.5 (11.4)	57.7 (3.8)	0.9
3mo SF12 mental difference	−0.6 (7.8)	3.5 (6.8)	0.2
12mo SF12 mental	60.4 (5.1)	50.4 (6.7)	**0.01**
12mo SF12 mental difference	3.6 (9.7)	−3.8 (6.2)	0.2

## Data Availability

Data is available upon request.
